# Height, Weight and Body Mass Index Percentiles of Children Aged 6-14 Years Living at Moderate Altitudes

**DOI:** 10.4274/jcrpe.559

**Published:** 2012-03-08

**Authors:** Ismail Malkoç, Mümtaz M. Mazıcıoğlu, Behzat Özkan, Meda Kondolot, Selim Kurtoğlu, Hakkı Yeşilyurt

**Affiliations:** 1 Ataturk University Faculty of Medicine, Department of Anatomy, Erzurum, Turkey; 2 Erciyes University Faculty of Medicine, Department of Family Medicine, Kayseri, Turkey; 3 İstanbul Medeniyet University Faculty of Medicine, Department of Pediatric Endocrinology, İstanbul, Turkey; 4 Erciyes University Faculty of Medicine, Department of Pediatric Endocrinology, Kayseri, Turkey; 5 Undersecretary of Ministry of Health, Department of Anatomy, Ankara, Turkey; +90 532 513 22 99ozkan.behzat@gmail.com

**Keywords:** weight, height, body mass index, adolescents, mid altitude, percentiles

## Abstract

**Objective:** Individuals living at high altitudes are reported to have lower stature and also a smaller chest size in relation to their stature. Altitude-related hypobaric hypoxia is considered to be the major cause of these alterations in growth, but adverse socioeconomic and/or other environmental conditions may also have a role in poor growth performance. This study was undertaken to provide growth data on children and adolescents living in a moderate-altitude area in Turkey.

**Methods:** The dataset of an anthropometric study conducted among a population living in a city at an altitude of 2000 meters was analyzed. A total of 1638 children and adolescents (871 males and 767 females) aged between 6 and 14 years were included in this study. The LMS method was used in the analysis and percentile values corresponding to the 3^rd^, 5^th^, 10^th^, 15^th^, 25^th^, 50^th^, 75^th^, 85^th^, 90^th^, 95^th^ and 97^th^ percentiles for height, weight and body mass index (BMI) were estimated. The results were compared with the measurements of children and adolescents living in areas of lower altitude in Turkey.

**Results:** Starting at ages 0-10 years, height, weight and BMI values of children and adolescents of both genders living at an altitude of 2000 meters were noticeably lower than those reported for their counterparts living in areas of lower altitude in Turkey.

**Conclusions:** The higher values for height, weight and BMI in children living in low-altitude areas can be attributed to altitude effect, but socioeconomic and microclimate effects cannot be discarded and further studies are needed.

**Conflict of interest:**None declared.

## INTRODUCTION

**Implications and Contribution**

Children living in different locations or under certain environmental conditions may need specific screening or follow-up criteria. Data on growth performance of children living in high-altitude areas will provide useful information for both screening and research purposes. 

Growth and development are complex physiological processes controlled or directed by several hormonal, environmental and genetic factors (1). Altitude is one of the environmental factors which may interfere with growth and development primarily by exposure to hypoxia (2,3). High altitude is defined as the level at which oxygenated hemoglobin concentration is lower than 90% and corresponds to altitudes of at least 2500 meters. Acute and chronic exposure to altitude-related hypoxia may lead to several permanent multisystemic problems (3,4). 

Exposure to altitudes between 1500 and 2500 meters (representing moderate altitude levels) has also been reported to cause various undesirable effects. 15-25% of individuals visiting these areas experience acute mountain sickness (5). Some other high altitude-related problems may be experienced at moderate altitudes as well (6).

The primary consequence of altitude-related alterations in growth is a decrease in linear growth and a smaller chest size relative to stature. However, the effects of the microclimate as well as that of socio-demographic variables are also pointed out as causes of growth problems in children living at high altitudes (7,8). 

While the effects of ethnic, socioeconomic and/or nutritional factors are well-documented, studies which analyze the effect of climate as a primary parameter interfering with growth are scarce (4,5,6). Our rationale for conducting this study was to produce weight, height and body mass index (BMI) percentile values in children and adolescents living at moderate altitude levels and to compare these values with the results of other growth studies in Turkish children. 

## METHODS

We used the dataset of an anthropometric study which was performed in 2007 on children and adolescents living in Erzurum, a city of an altitude of 2000 meters in Turkey (9). A total of 1638 children and adolescents (871 males and 767 females) aged between 6 and 14 years were included in this study. The selection of the subjects was done after stratification according to their socioeconomic level and age, using the indicators of the Turkish Statistical Institute (10). Children who had chronic or systemic diseases that may interfere with growth were excluded from the study. The ethical approval for the study was obtained from the institutional review board of the Ataturk University in Erzurum.

The Harpenden stadiometer, a device calibrated to measure height with an accuracy of 0.05 cm, was used for height measurements. All subjects were measured barefooted and lightly clothed. Weights were measured using a standard beam scale, sensitive to 0.1 kg (Tefal Ultraslim). Weight and height measurements were repeated twice, and the average value was recorded. All measurements were taken between 8 and 12 am to avoid errors due to within-day variability (11). BMI was calculated according to the standard equation [weight (kg)/ height (m)^2^].

The LMS method was used to fit smooth centile curves to the reference data (12,13). With this method, percentiles based on age-specific Box-Cox power transformations that are used to normalize the data can be expressed. The final curves are produced by three smooth curves representing L (Lambda; skewness), M (Mu; median) and S (Sigma; coefficient of variation). In this present study, the percentile values were estimated by the LMS Chart Maker Proversion 2.3 software program (The Institute of Child Health, London) and the centile curves (3^rd^, 5^th^, 10^th^, 15^th^, 25^th^, 50^th^, 75^th^, 85^th^, 90^th^, 95^th^ and 97^th^) were constructed using Microsoft Office Excel® version 2003. 

We compared the 3^rd^, 50^th^, and 97^th^ percentile values for height, weight and BMI obtained in this study in Erzurum with the cross-sectional data from Turkish children residing in Kayseri (altitude of 1050 meters) (14). We also made a three-way comparison of 50^th^ percentile values for height, weight and BMI obtained in this study (Erzurum; 2000 meters) with the data from children living in Istanbul (seaside level) and Kayseri (1050 meters) (14,15). Descriptive statistics for each whole year (e.g., 6.00-6.99 y, etc.) within gender were calculated using SPSS version 13.0 (Illinois, Chicago, USA).

## RESULTS

Tables 1, 2, 3 present LMS values and 3^rd^, 5^th^, 10^th^, 15^th^, 25^th^, 50^th^, 75^th^, 85^th^, 90^th^, 95^th^ and 97^th^ percentile values for weight, height and BMI by age and gender in 6-14 years old children and adolescents living in Erzurum at an altitude of 2000 meters. Figures 1a, 1b, 1c, 1d, 1e, 1f depict the 3^rd^, 50^th^ and 97^th^ percentiles for height, weight and BMI in both genders in Erzurum and Kayseri subjects. 1a, 1b, 1c, 1d, 1e, 1f compare 50^th^ percentile values in both genders in children from three cities of different altitudes (Erzurum - 2000 meters, Kayseri - 1050 meters, Istanbul - seaside).

## DISCUSSION

Hypobaric hypoxia due to high altitude leads to physiological adaptations in body functions and composition. These adaptation efforts may lead to physical alterations, pertaining to body fat, body weight, BMI and chest dimensions (16). These changes may be transient or permanent depending on time of exposure to altitude, level of altitude, speed of ascent and personal characteristics (5). In view of reports indicating that hypobaric hypoxia may cause alterations in growth even at moderate altitudes, we designed this preliminary study to compare the growth indicators of children living in a moderate-altitude area with those of children of the same geographic region but living in areas of lower altitude (Kayseri, Turkey and Istanbul, Turkey). The lowest altitude where hypobaric hypoxia is reported to occur is 900 meters (17).

There are studies indicating that children living at altitudes of 3000 meters or over are lighter and shorter when compared with their counterparts who are born and brought up at lower altitudes (18,19). However, reports also indicate that other than high altitude and hypobaric hypoxia, adverse microclimate conditions (heat, humidity, wind levels) as well as individual characteristics may also cause poor growth performance in these children (20,21).

In this preliminary study, based on the premise that alterations in growth and development may also occur in areas of moderate altitude, we produced age- and gender-specific percentiles for children and adolescents aged between 6 and 14 years and living in a moderate-altitude area (Erzurum) in Turkey. 

We compared the 3^rd^, 50^th^ and 97^th^ percentile values for height, weight and BMI of these children living at a moderately high altitude (2000 meters) with those of their counterparts living in a lower altitude (1050 meters) area and found that the values were noticeably lower in the higher altitude group in both genders, starting at ages 9-10 years (Figures 1a, 1b, 1c, 1d, 1e, 1f). 

We also compared the 50^th^ percentile values obtained from these children living in an area of moderate altitude with those of children living at 1050 meters and at seaside level. Although there are differences in the design of these three studies, we believe that a comparison of 50th percentile values would be beneficial to a discussion on growth at different altitude levels Figures 2a, 2b, 2c, 2d, 2e, 2f). The above-mentioned comparisons also showed no noticeable differences in growth at younger ages, but a decline in 50th percentile values in children reared in the moderately high altitude area as compared to the other two groups, starting at ages 9-10 years.

The major limitations of our study may be the relatively small sample size and the lack of information on inter-observer and intra-observer differences.

In conclusion, the current study provides height, weight and BMI percentiles of Turkish boys and girls aged between 6 and 14 years living in a moderately high altitude area in Turkey. The results indicate that these children show a relatively stunted growth after ages 9-10 as compared to children living at lower altitudes in Turkey. These results point to the possibility of an altitude effect, but differences caused by socioeconomic and microclimate characteristics cannot be dismissed. 

## Figures and Tables

**Table 1:**
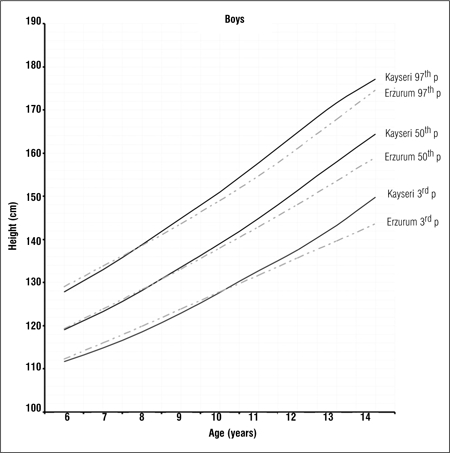
LMS values and 3^rd^, 5^th^, 10^th^, 15^th^, 25^th^, 50^th^, 75^th^, 85^th^, 90^th^, 95^th^ and 97^th^ percentiles for height in Erzurum boys and girls

**Table 2:**
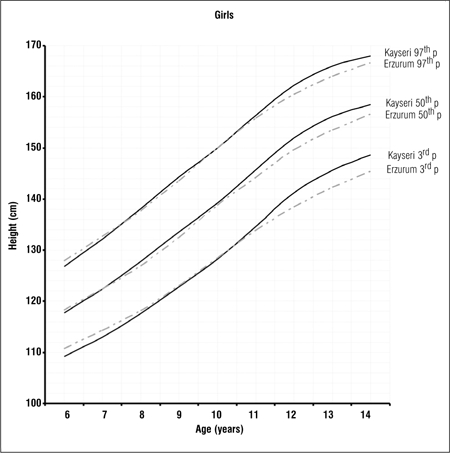
LMS values and 3^rd^, 5^th^, 10^th^, 15^th^, 25^th^, 50^th^, 75^th^, 85^th^, 90^th^, 95^th^ and 97^th^ percentiles for weight in Erzurum boys and girls

**Table 3:**
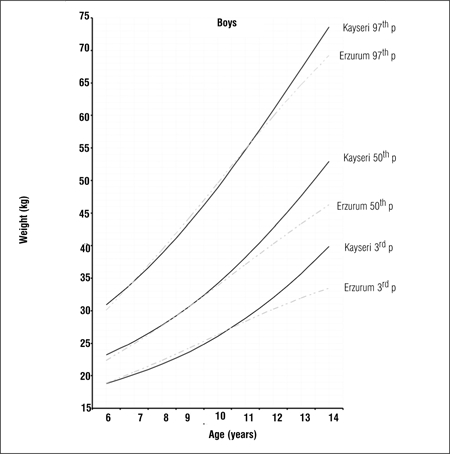
LMS values and 3^rd^, 5^th^, 10^th^, 15^th^, 25^th^, 50^th^, 75^th^, 85^th^, 90^th^, 95^th^ and 97^th^ percentiles for BMI in Erzurum boys and girls

**Figure 1a:**
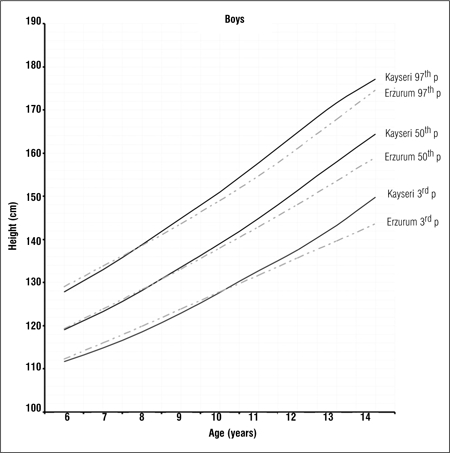
3^rd^, 50^th^ and 97^th^ percentile values for height in Erzurum and Kayseri boys

**Figure 1b:**
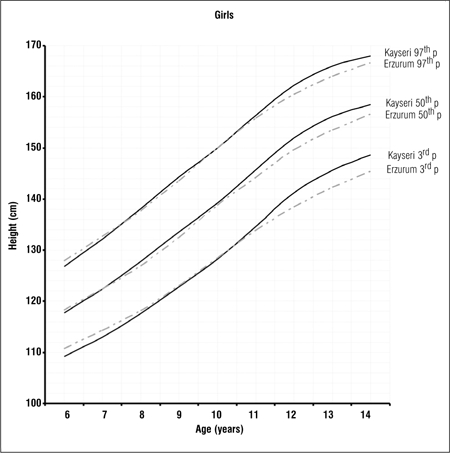
3^rd^, 50^th^ and 97^th^ percentile values for height in Erzurum andKayseri girls

**Figure 1c:**
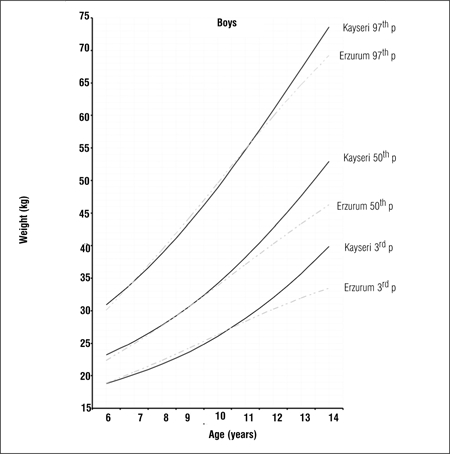
3^rd^, 50^th^ and 97^th^ percentile values for weight in Erzurumand Kayseri boys

**Figure 1d:**
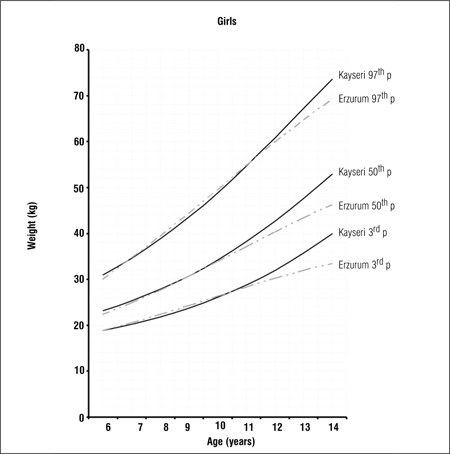
3^rd^, 50^th^ and 97^th^ percentile values for weight in Erzurumand Kayseri girls

**Figure 1e:**
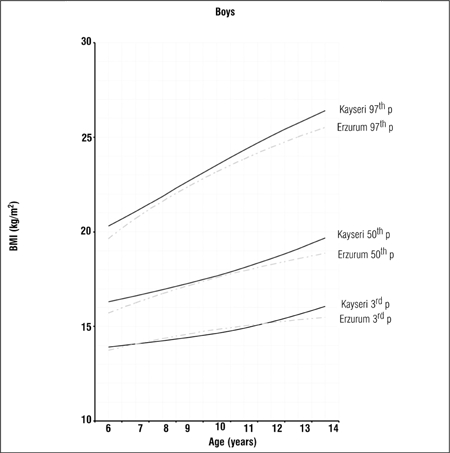
3^rd^, 50^th^ and 97^th^ percentile values for weight in Erzurumand Kayseri boys

**Figure 1f:**
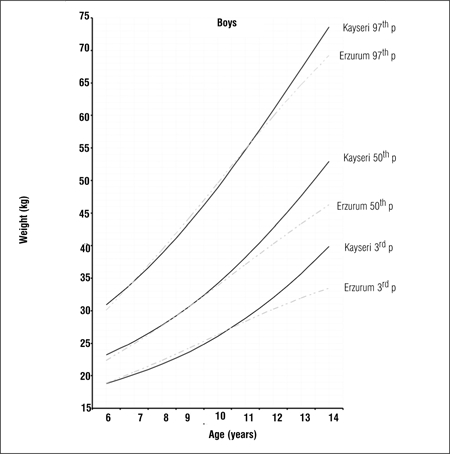
3^rd^, 50^th^ and 97^th^ percentile values for BMI in Erzurum and Kayseri girls

**Figure 2a:**
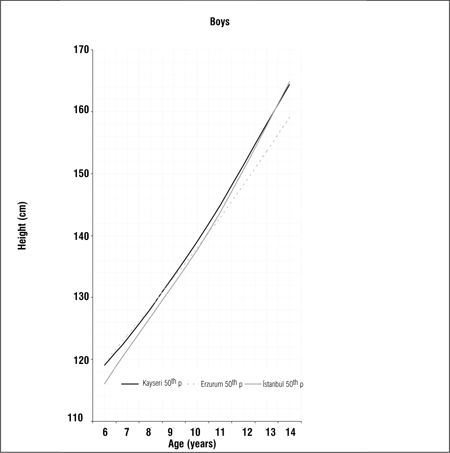
50^th^ percentile values for height in Erzurum, Kayseri and Istanbul boys

**Figure 2b:**
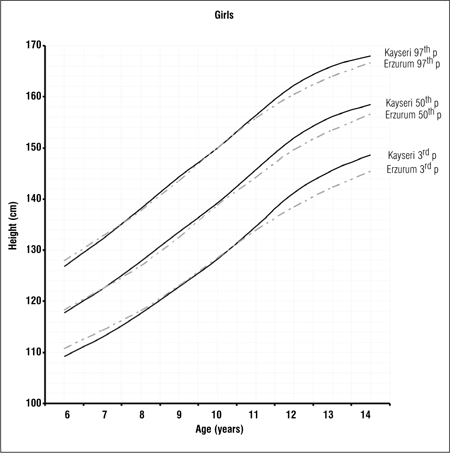
50^th^ percentile values for height in Erzurum, Kayseri and Istanbul girls

**Figure 2c:**
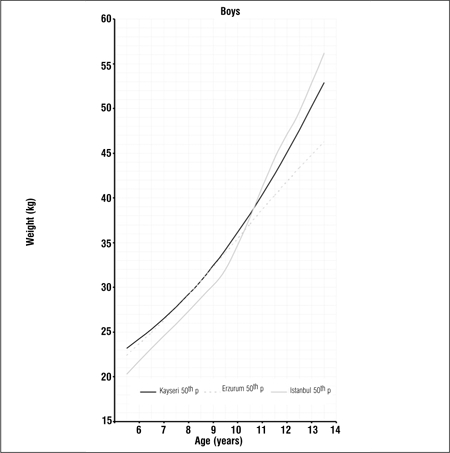
50^th^ percentile values for weight in Erzurum, Kayseri and Istanbul boys

**Figure 2d:**
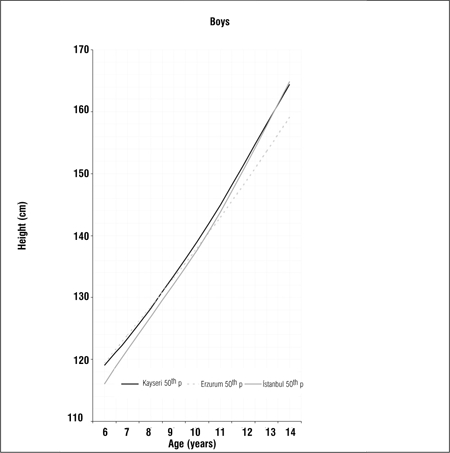
50^th^ percentile values for weight in Erzurum, Kayseri and Istanbul girls

**Figure 2e:**
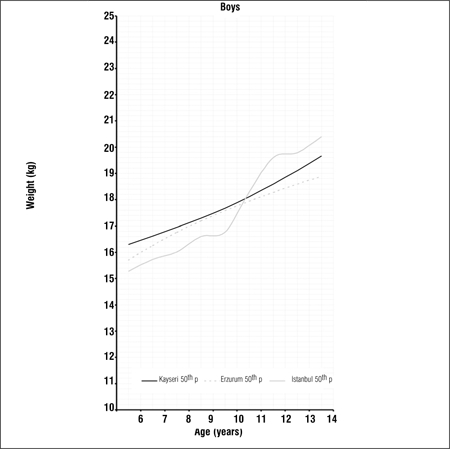
50^th^ percentile values for BMI in Erzurum, Kayseri and Istanbul boys

**Figure 2f:**
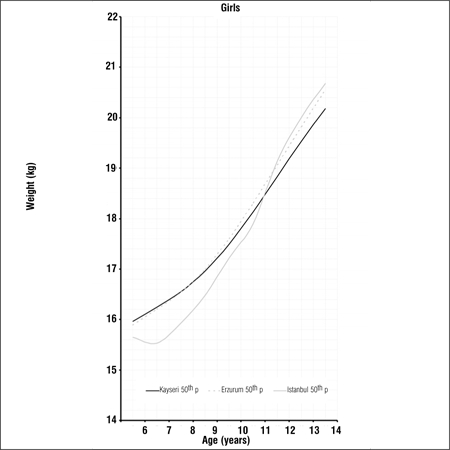
50^th^ percentile values for BMI in Erzurum, Kayseri and Istanbul girls
